# Critical Appraisal and Hazards of Surface Electromyography Data Acquisition in Sport and Exercise

**DOI:** 10.5812/asjsm.34868

**Published:** 2010-06

**Authors:** Jan Pieter Clarys, Aldo Scafoglieri, Jonathan Tresignie, Thomas Reilly, Peter Van Roy

**Affiliations:** 1 Department of Experimental Anatomy (EXAN-LK), Vrije Universiteit Brussel, Belgium; 2 Research Institute for Sport and Exercise Sciences (RISES), Liverpool John Moores University, UK

**Keywords:** SEMG, IEMG, Normalization, Electrode localization, EMG/force

## Abstract

The aim of this critical appraisal and hazards of surface electromyography (SEMG) is to enhance the data acquisition quality in voluntary but complex movements, sport and exercise in particular. The methodological and technical registration strategies deal with telemetry and online data acquisition, the placement of the detection electrodes and the choice of the most adequate normalization mode.

Findings compared with the literature suggest detection quality differences between registration methods and between water and air data acquisition allowing for output differences up to 30% between registration methods and up to 25% decrease in water, considering identical measures in air and in water. Various hazards deal with erroneous choices of muscles or electrode placement and the continuous confusion created by static normalization for dynamic motion. Peak dynamic intensities ranged from 111% (in archery) to 283% (in giant slalom) of a static 100% reference. In addition, the linear relationship between integrated EMG (IEMG) as a reference for muscle intensity and muscle force is not likely to exist in dynamic conditions since it is muscle – joint angle – and fatigue dependent. Contrary to expectations, the literature shows 30% of non linear relations in isometric conditions also.

SEMG in sport and exercise is highly variable and different from clinical (e.g. neurological) EMG. Choices of electrodes, registration methods, muscles, joint angles and normalization techniques may lead to confusing and erroneous or incomparable results.

## INTRODUCTION

The aim of this work (e.g. technical notations) is to provide more clarity in the variability of surface electromyography (SEMG) when applied to sport and exercise. A critical appraisal of this SEMG variation and description of hazards in measurements and choices should improve the methodological quality of data acquisition of complex dynamic voluntary skills and movements. Background knowledge of trial and error, problems and differences of EMG in general and SEMG in particular are the bases of consensus on key items that enable exchange, reproduction and comparison of the electrical activities of complex dynamic and isometric contractions.

Man has shown a perpetual curiosity about the origins of locomotion in human and other creatures. Amongst the oldest scientific experiments known to us, we must name the detection of electricity and function of muscle^[1, 2, 3]^.

The detection of the electrical signal of the human and animal muscle emanates from long before Galvani who took the credit for it^[4]^. Jan Swammerdam already showed the mechanics of muscular contraction to the duke of Tuscany in 1658^[5]^. Even if ‘Electrology or localized electrisation’ – the original terminology for EMG – could be the oldest (if not the oldest) bio-scientific detection and measurement technique, it has remained for very long and until a few decennia ago, a rather ‘supporting’ measurement with limited discriminating qualifications always to be used in conjunction with other measuring methods.

Developments in signal processing, acquisition- and analysis systems upgraded SEMG into a problem solving, problem detecting and problem discriminating scientific discipline.

The reason why EMG needed such a long time to obtain this status is assumed to be related to the fact that EMG took 3 distinct different directions in the course of its development; each with various approaches and analytic techniques^[6, 7]^.

The clinical EMG is majorly a diagnostic tool. The kinesiological EMG (including SEMG) is merely a system to study function and co-ordination, while the (fundamental) EMG itself, deals with single fiber- and motor unit action potentials (SFAP and MUAP) and the related time – frequency domain. Depending on the user (a physician, an anatomist, an ergonomist, a physiologist, an engineer, a physiotherapist, a neurologist), one encounters independent improvements in registration technology, different approaches of data acquisition and various but specific graphic representations, analytic techniques and signal treatments software.

All this has resulted into a number of applications in neurology, neurophysiology, neurosurgery, bioengineering, Functional Electro Stimulation (FES), orthopaedics, occupational biomechanics and –medicine, rehabilitation and physical therapy, sport medicine and sport science. This dissemination of knowledge about the neuromuscular systems both in normal and the disabled was already predicted (and in part described) by Duchenne de Boulogne (1872)^[8]^.

Electromyography became a diagnostic tool for studies of muscle weakness, fatigue, pareses, paralysis, conduction velocities and lesions of the motor unit or for differential neurogenic and myogenic problems. FES developed as a specific rehabilitation tool. Almost in parallel and within the expanding area of EMG, a particular speciality developed wherein the aim is to use EMG for the study of muscular function and co-ordination of muscles in different movements and postures.

The application areas of kinesiological EMG and therewith SEMG can be summarized as follows: studies of normal muscle function during selected movements and postures; studies of muscle activity in complex sports; occupational and rehabilitation movements; studies of isometric contraction with increasing tension up to the (relative) maximal voluntary contraction; evaluation of functional anatomical muscle activity (validation of classical anatomical functions); co-ordination and synchronization studies (kinematic chain); specificity and efficiency studies of training methods; fatigue studies; the relationship between EMG and force; the human-machine interaction; studies on the influence of material on muscle activity; vocational loading effect studies related to low back pain and arthrokinematics.

Within these various applications, the recording system (e.g. the signal detection, the volume conduction, the signal amplification, impedance and frequency responses, the signal characteristics) and the data-processing system (e.g. rectification, linear envelope and normalization methods) are going hand in hand with a critical appraisal of choices, limits and possibilities^[7]^.

**Fig. 1:**
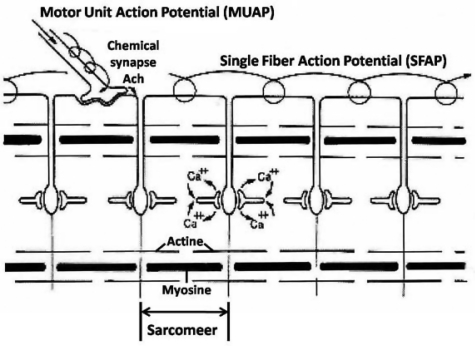
When a skeletal muscle fiber is activated by a MUAP, a wave of electrical depolarization, referred to as an SFAP, travels along the surface of the fiber

Recently Merletti et al. (2009) presented the state of the art of the technology of detection and conditioning systems for SEMG, with a focus on electrode system technology, electrode classification, impedance, noise, transfer function, spatial filtering and on SEMG multi-electrode grids and multi channel amplifiers^[9]^.This review, however, majorly related to (intra- and extracellular) SFAP and MUAP (Fig. 1), was described earlier as the fundamental part of kinesiological EMG.

**Fig. 2:**
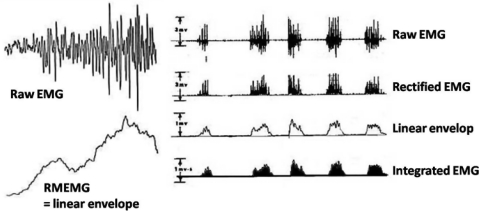
The basic EMG signals used in dynamic and complex sport, exercise and occupational activities

The purpose of this work is to consider the activity of a whole muscle (e.g. collection of SFAP's) or functional muscle groups including the specific associated treatment of the signal, e.g. full wave rectification of the raw EMG, averaging into a linear envelop and integrate (IEMG) to obtain a value of muscular intensity over time (Fig. 2).

Following the criteria of the International Society of Kinesiological Electromyography, SENIAM and the Journal of Electromyography and Kinesiology, it is recommended to report the upper cut-off frequency, the lower cut-off frequency and the type of filter used in the amplifiers. If a direct coupled amplifier is used, the input impedance and input current should also be reported^[6]^. The type and material of the electrodes, the space between the contacts, the site and the preparation of the skin should also be documented. With respect to the processing of data, it is important to mention not only the use of raw EMG, IEMG, linear envelope, mean rectified EMG (MREMG), together with the synchronization system and the normalization technique used; such as normalized to maximal voluntary contraction (MVC) or to 50% of the average of three MVCs, or to the highest peak (per movement or per subject) or to the mean of the subject ensemble average.

The linear envelope is the qualitative expression of the rectified and eventually averaged signal within a window choice; independent of its purpose, this linear envelope can be smoothed. It should equally be clear that once smoothing is started, integration is no longer possible and it is unwise to use ‘intensity’ or ‘activity level’ in this case. Integration refers to the surface under the non-smoothed but rectified signal, to express the phenomenon of ‘muscular intensity’.

### Telemetric, on-line and remote on-line registration approaches

Over the years, the improvement of devices for registering the EMG signal and the evolution of methodological approaches to both EMG data acquisition and computerized pattern analysis have been valuable for bio-engineering, occupational- and sport medicine, physiotherapy, sports biomechanics, and also eventually for trainers and coaches. Since the end of the 1960s there has been a development in miniaturized telemetric devices for monitoring complex human movements remotely. Especially for kinesiological purposes, the telemetric devices have recently been changed from two-channel registrations to eight or more-channel systems.

There are obvious and numerous advantages to telemetric measurement of muscular activity, although some difficulties may be encountered in field circumstances. For example, it is difficult to link more than two or three transmitters in parallel due to the limited free radio wave possibilities at present, in particular if working on location. Secondly, since the beginning of the research employing telemetric EMG breaks in transmission, atmospheric conditions, static or other disturbances have never been truly controllable and this has not changed today^[10]^.

In order to measure muscle activity in complex sports movements in the laboratory or field studies, different features are taken into consideration:

- The EMG data acquisition system with its electrodes should allow total freedom of movement for the subject – in other words, movements without additional resistance.
- The set-up should allow adaptation to the characteristics of the field and movement circumstances, in different situations. It should be applicable to activities in sports such as swimming, skiing, archery, cycling and so on, and to the working environment of health-care professionals. It should accommodate long-term activity and movement over large distances and allow continuous measurements (up to tape limits).
- It should be possible to monitor at least six to twelve muscles simultaneously.
- The combined registration and data acquisition setup should be user-friendly.

**Fig. 3:**
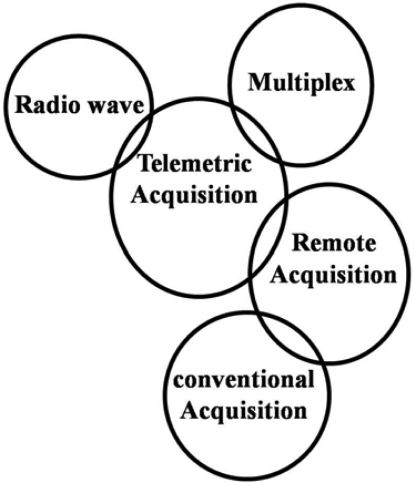
SEMG data acquisition systems to be used in sport, exercise and occupation

A multichannel FM recorder, ‘active’ electrodes, a regulation-amplification unit and different synchronization modes need to be integrated into one system with different possibilities in order to allow such a combination. This integration was done in both a ‘conventional on-line’ and ‘remote’ configuration (Fig. 3)^[11]^.

In practical terms, a conventional on-line system will connect the (active pre-amplified) electrodes of the athlete with a wire to the (additional) amplification and data acquisition system. This methodological choice is suggested for all movements within a limited area and/or with limited displacement of the athlete (e.g. shooting sports, treadmill running, cycling on roles, etc…). Most sports, however, need distance (ski, rowing, cycling, track running, etc…).

Two methodological solutions are at hand: (i) A telemetric system (with amplifiers on the subject) with radio wave characteristics (e.g. transmitter on the subject-receiver at distance) or with a multiplex system. (ii) A remote (on-line) system. Electrodes are amplified on the subject and signals are registered on miniaturized recording systems. The whole system is on-line and carried by the subject. A distance control system serves to start and stop the registration system remotely (Fig. 3).

The method of choice is the remotely control on-line system carried by the athlete because of its higher reliability. Measurements (on the M. biceps and M. brachioradialis) with one pair of electrodes per muscle, identical amplification between muscle and registration unit taken simultaneously with a telemetric and a remote (on-line) system on subjects (N=9) executing identical isometric and dynamic functions indicated loss of electricity (IEMG) with a telemetric system^[12, 13]^.

### Electrodes, water and air data acquisition

The choice is easily made among the most commonly used sensor (e.g. passive surface electrode Ag/AgCL – ø10mm), the active pre-amplified surface electrodes, the intra muscular needle and wire electrodes. The active electrode being the most reliable for complex dynamic sport and exercise movement^[7, 10]^.

The advantage of the active electrode over the classic passive electrode is decline of erroneous registrations. This feature has become important since we have found that, despite thorough precautions (different taping and plastic varnish for protecting the electrodes), water does decrease the detectable electrical output of human muscle. In other words, an imaginary identical intensity will produce more electricity in air than in water. The implications for using EMG in aquatic environments have been reviewed elsewhere^[12, 13]^.

Recently, there has been a renewed interest in comparing the reproducibility of EMG under immersion and on land, mostly in a EMG/force relation. Pöyhönen et al. (1999) observed that MVC force output and muscle intensity significantly decreased during immersion compared to the same motion on land^[14]^. This significant signal decrease ranged between 11 and 35%, depending on the muscle studied^[15, 16]^.

Rainoldi et al. (2004), Veneziano et al. (2006) and Masumoto and Mercer (2008) experimented with waterproofing electrodes and obtained corresponding results. Maximal effective waterproofing can decrease the water and on land differences to zero^[17, 18, 19]^.

**Fig. 4:**
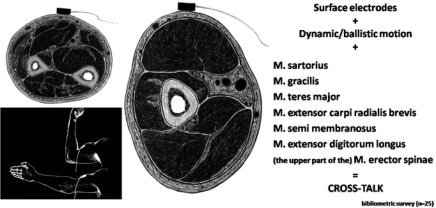
If the muscle belly surface is too small and/or of the detection electrode is placed on a different location than the midpoint of the contracted muscle in dynamic situations, the investigator will have to deal with cross-talk

Both in water and on land, in particular for dynamic registrations, the placement of the detection and the reference (ground) electrodes are of great importance. They do not correspond to the SENIAM recommendations which focuses merely on amplitude and frequency characteristics, timing properties and MUAP width and shape^[20]^. Dynamic SEMG in sport and exercise is looking at a much wider picture of whole muscle contraction and relaxation (e.g. inter-muscular and inter-individual synchronization). Therefore, localizing the detection electrode according to standard osteologic reference points is to be avoided. In a complex sport and exercise activity combining concentric, eccentric and co-contractions, the muscle belly will travel continuously up and down through the contraction/elongation axis and thus under the skin. If the detection electrode is located differently from the midpoint of the contracted muscle, chances will be considerable and at some levels of the displacement, the detection electrode loses the muscle under the skin (Fig. 4).

At the same time, not all muscles are suitable for SEMG. If the muscular surface is less than 10cm^2^, possibility of losing the muscle to be detected under the electrode, increases significantly leading almost automatically to cross-talk (Fig. 4).

Cross-talk is the detection of myo-electric activity of neighboring muscles instead of, or in combination with the originally studied muscle. Not all cross-talk will lead to erroneous registrations. If the neighboring muscles are active as a function group within the complex movement, the summation of motor unit contribution can alter the intensity, not the function. Cross-talk is to be considered as a side effect inherent to SEMG^[21]^.

In general, it is agreed that electrodes should be placed on the muscle from which a good and stable SEMG signal can be recorded. The SENIAM recommendations for that matter, take into account the location of motor points, muscle tendons and osteologic references^[20]^. These issues become irrelevant if the midpoint of the contracted muscle is chosen^[6, 7, 22]^.

### Critical appraisal limitations and hazards with SEMG

Most activities in sport and exercise settings involve complex movement patterns that are often complicated by external forces, impacts and the equipment used during the movement. An electromyogram (or its derivates) is the expression of the dynamic involvement of specific muscles within a determined range of that movement. Mostly SEMG is used to investigate the activity of a series of muscles, seldom just one or two. The choice of these muscles is based either on practical knowledge of the skill or on basic anatomy. Thorough knowledge of both sport and kinesiology are essential. It is important to note that the selection of muscles for SEMG measurements requires careful considerations. Some of these choices can lead to erroneous registration, sometimes without being noticed by peer reviewers^[6]^.

Various researchers have placed surface electrodes on the M. sartorius, the M. gracilis and the M. teres major. Measuring the SEMG of these muscles under static conditions creates little or no problems, but under complex dynamic conditions, the sartorius and gracilis muscles disappear from under the electrodes as does the M. teres major, especially during arm motion above 90° abduction. It is therefore uncertain which muscles have contributed to the EMG patterns presented. Other research groups have selected for their studies the M. extensor carpi radialis brevis. This muscle has a very small superficial ‘strip’ accessible under the skin. The EMGs of this muscle are dubious and may give more information about the M. extensor digitorum. The same problem arises during measurement of the M. semimembranosus (under the M. semitendinosus); although the superficial muscle belly parts may seem sufficient, the combination of displacement of the superficial M. semitendinosus with a lack of functional surface again gives different information from what was expected (e.g. the cross-talk phenomenon).

The use of wire electrodes does not necessarily have this problem, although measuring the M. subscapularis in this way – especially during front crawl in swimming and during golf swing movements, for example, – is questionable. The point of view of the anatomist who is confronted with these situations in the dissection room and palpation classes should not be discounted. The competent anatomist will not select the M. sacrospinalis, but instead chooses the M. erector spinae for measurement. One group, however, reported measuring the SEMG of the M. tibialis posterior during skiing. Clarys (2000) assumed that this was a printing error^[6]^.

The reference or ground electrode is another matter. It is a common knowledge that the reference electrode is placed on electrical inactive tissue (e.g. bone). The idea is to minimize (common mode) disturbances. The literature suggests a series of preferred locations, e.g. the wrist bones, the crista iliaca, the tibia, the processus spinosus of C_6_-C_7_ and the sternum. For complex dynamic movements the sternum and the os frontalis are the choices of preference.

**Fig. 5:**
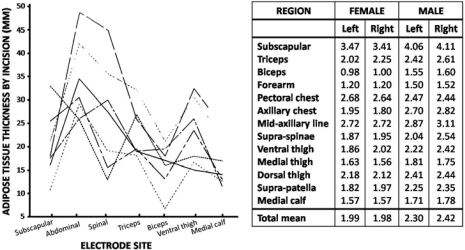
Mean skin thickness per region, left and right, males and females and adipose tissue (AT) depth at a selection of classic electrode locations. (Data from a cadaveric study^[23]^)

Good skin preparation for the better fixation of the electrodes consists of shaving, sandpapering and cleaning. But it is only expected to have indirect effects on the SEMG signal quality. Proper skin preparation is necessary to reduce skin (and subcutaneous tissue) impedance. Skin impedance phenomena are common knowledge but too little interest is given to the related human Body Composition (BC), the variation of skin thickness and subcutaneous adipose patterning^[23]^. Figure 5 clearly indicates the extension of this variability but no direct relation has been studied between SEMG and BC at different locations, yet.

### Normalization … the choice of the better reference

No two EMG profiles that result in the same action are ever identical, because of the change in the actual motor units controlling the activity. Due to this known variability of the EMG signal, not only between subjects but also between different trials, different normalization techniques have been developed to reduce variability. In general, the EMG of maximum effort or the highest EMG value has been selected as the normalizing factor. In the main, the subject is asked to perform a MVC of the muscle (groups) being studied. This amplitude, either raw or rectified, over time is then used as a reference value (e.g. 100%). The use of the MVC reference is very popular and is acceptable in all static applications. For all dynamic activities the use of an isometric reference is questionable. Several investigators have recently reported EMG values in dynamic activities that exceeded the maximal isometric effort (Table 1).

**Table 1:** Dynamic SEMG intensities compared to 100% static MVC in different sports studies and at different velocities in swimming^[6]^

	Examples	Up to	M. Triceps Brachii (% of 100% MVC)		
				Maximum (N=1)	X total (N=60)
**Lewillie, 1973**	Back Stroke	142%	**Crawl**		
	Breast Stroke	120%	**Slow**	104	86
	Dolphin	160%	**Normal**	114	96
			**Sprint**	124	110
**Clarys et al., 1983**	Crawl	138%	**Breast Stroke**		
	Sculling	195%	**Slow**	56	20
			**Normal**	96	60
**Jobe et al., 1984**	Baseball pitching	226%	**Sprint**	120	80
			**Back Stroke**		
**Clarys et al., 1990**	Archery (90m)	111%	**Slow**	106	72
**Hosea et al., 1990**	Golf swing	115%	**Normal**	114	80
			**Sprint**	142	96
**Clarys et al., 1994**	Special slalom	180%	**Dolphin**		
			**Slow**	102	80
**Hintermeister et al., 1997**	Giant slalom	283%	**Normal**	114	80
			**Sprint**	160	104

Therefore, other normalization techniques have been developed in kinesiological EMG especially for sport exercise and occupation, e.g. normalization to the highest peak activity in dynamic conditions, to mean integrated EMG (ensemble average), to EMG per unit of measured force (net moment), and so on.

In an extensive review of sport specific and ergonomic studies using EMG, the missing information mostly concerned the issue of normalization. In the majority of both sport and occupational studies in which a normalization technique is mentioned, the MVC technique has been used. This approach, however, is unreliable in all dynamic situations for several reasons^[6]^:

- Different maxima may be observed within the same subject repeating at different occasions, the same ‘maximal’ but isometric effort
- Different maxima are observed at different angles of movement, both in eccentric and concentric movement modes
- Additionally, the question of linearity may arise when the values measured during isotonic dynamicballistic- complex sports movements (or heavy lifting tasks) exceed the 100% MVC. For example, Clarys et al. (1983) found dynamic percentages in swimming up to 160%, while Jobe et al. (1984) reported up to 226% of MVC in baseball pitching and Hintermeister et al. (1997) even 283% of MVC reference in a giant slalom (Table 1)^[24, 25, 26]^.

It seems reasonable to suggest that a statically obtained EMG, such as MVC, cannot be an appropriate reference for dynamic EMG. In other words, most problems disappear when the proper normalization techniques are used. The data must be standardized for the cycle time and the amplitude. Several types of amplitude normalization are in use. All of them work and are valid, but only if applied correctly. The important thing is to choose a normalization technique, apply it rigorously, compare the reported results with your clinical estimate of the patient's or athlete's performance and adjust either your perception of performance or the EMG technique to achieve agreement between the EMG and actual performance (Table 2).

**Table 2:** Frequently used normalization techniques in sport, exercise and occupation

For STATIC purposes	For DYNAMIC purposes
MVC (pre-post test) max. effort Average of three 50% MVC EMG amplitude per unit isometric moment of Force Relative Voluntary Contraction (RVC) average of three isometric contractions overtime (20sec) with a known load (10 to 20 kg range)	Highest peak (amplitude) overtime (sec) within a movement series (within the same subject) The highest activity (IEMG) of different ensemble averages

Kinesiological reasoning and basic biomechanics tell us that statically obtained EMG cannot create a reference for dynamic-, ballistic- and complex EMG. It would be physiologically and biologically incorrect.

### Activity level in terms of IEMG and force, strength, torque

**Fig. 6:**
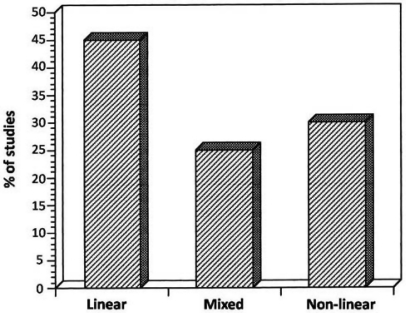
Distribution (%) of IEMG – Force relations in isometric conditions

In isometric work the IEMG signal – being the expression of muscular intensity – is by many assumed to be proportional to the force generated by the muscle action. Muscle intensity however, even isometric is not always linearly related to the force, if such relation does exist!? A scanning of 31 references dealing with the relation between IEMG (mV) and force (N or kg) reveal a majority, indeed of linear relationships between IEMG and force. But a considerable percentage of mixed (e.g. not perfectly linear) and of non-linear relationships were found (Fig. 6).

**Fig. 7:**
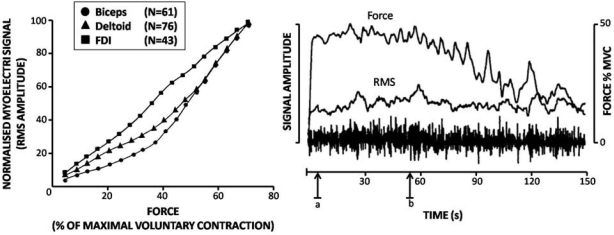
Linearity can deviate depend on muscle function (left)^[27]^, and linearity of the EMG/force relationship can change over time (right)^[31]^

The mixed group is or can be most confusing, e.g. if EMG data are not supported by other variables. The degree of linearity is influenced by the muscle type and its function^[27]^ or can be changing within the same isometric contraction from a linear to a non-linear relation over time (Fig. 7). Raw EMG and the linear envelop (RMEMG) can stay unchanged, but force will decrease over time. If we want to maintain that force, although one cannot hold it, the EMG will increase^[1]^.

Under dynamic conditions, the relationship is not so simple due to the changing force-torque characteristics at different phases of movement. In very fast, ballistic-type motions, the EMG signal demonstrates alternating bursts of activity in both agonist and antagonist muscles. In such movements the EMG is triphasic in activity^[28]^. A first burst of EMG activity is evident in the agonist, followed by signals from the antagonist. The first burst of activity from the agonist represents the propulsive force that initiates limb motion whereas the first activity in the antagonistconcludes with deceleration of the limb. The second phase of activity in the agonist determines the final positioning of the limb.

During eccentric muscle actions, the tension generated can exceed that during isometric action which in turn is greater than during a concentric action when the same muscle shortens in length. Despite the high EMG activity during an eccentric action, the energy expended may be quite low since fewer motor units may be recruited. It is during eccentric muscle actions that muscle damage may occur. Leakage of creatine kinase through the muscle membrane into the blood is evident following eccentric work and the ensuing soreness peaks 48-72 hours later. The phenomenon of ‘delayed onset muscle soreness’ may also be linked to an inflammatory response due to micro-trauma within the muscle and its connective tissue. The damage occurs due to the forces involved in stretching the muscles which are resisted by the contractile components^[7, 29]^.

Eccentric actions are dominant in weight lowering activities compared to the predominance of concentric (and isometric actions) in lifting activities.

The electromyographic activity can be seen as a neuromuscular response to match the biomechanical requirements. The EMG signal information can be used in different ways depending on the question at hand. A basic question is whether we are interested in forces and torques (biomechanics) or muscle activation (physiology). Both approaches have sport and occupational medical relevance. However, an investigator should decide which approach is the most suitable for the actual question at issue since the chosen approach is closely linked to the choice of calibration strategy and interpretation of results^[29]^.

If we are interested in forces and torques, we have to establish a calibration curve between SEMG, IEMG in particular and force or torque^[30]^. This relationship involves several error sources which too often have been overlooked. With this biomechanical approach, fatigue effects are considered as confounders since they alter the EMG-force relationship.

**Fig. 8:**
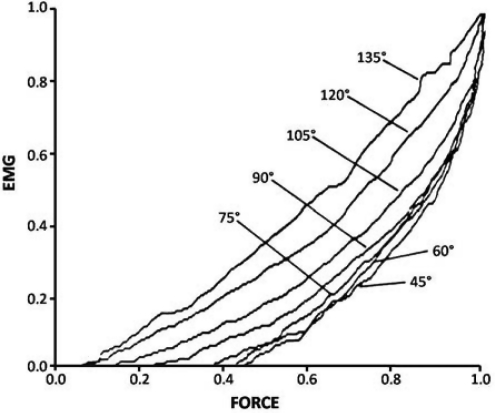
The joint angle during sport and exercise will seriously influence the EMG/force relationship

In addition, every joint angle within a dynamic complex muscle activity will influence the EMG/force relation making strength assumption from EMG data almost impossible and biologically irrelevant (Fig. 8)^[ 31 ]^.

## CONCLUSION

SEMG in sport, exercise and occupation is a clear subtopic of kinesiological EMG and its experimental approach including signal treatment is different from SFAP and MUAP studies (e.g. intra and extra cellular).Kinesiological EMG, e.g. as a functional and co-ordination tool, is different from clinical or neurological EMG which is merely a diagnostic tool.

Sport and exercise need data acquisition systems that allow total freedom of motion. The on-line in combination with a remote registration system accommodates best long term and distance or surface demanding movements.

Water and telemetry may be at the origin of signal decrease (loss) while muscle displacement under the detection electrode will create cross-talk or will measure other activities than assumed. The localization of the electrode on the midpoint of the contracted muscle which has to be superior to 10cm2 will eliminate a maximum of cross-talk.

As to the better normalization technique in sport and exercise, MVC references are perfect for all static circumstances, but must be avoided in dynamic situations.

IEMG being the intensity signal is not per se similar to force, strength and torque e.g. µV is not the same as N or kg.

Skin impedance is a well known signal treatment factor, but the variability of skin thickness and subcutaneous layer patterning deserves more study.
